# Editorial: Improve metabolic and autoimmune diseases by regulating gut microbiota

**DOI:** 10.3389/fimmu.2022.1091805

**Published:** 2022-11-29

**Authors:** Yongbo Kang, Peng Ren, Xiaoyu Kuang, Liping Li, Xing Kang, Ying Yang

**Affiliations:** ^1^ Department of Endocrinology, Affiliated Hospital of Yunnan University, Kunming, China; ^2^ Department of Microbiology and Immunology, School of Basic Medical Sciences, Shanxi Medical University, Taiyuan, China

**Keywords:** gut microbiota, metabolic diseases, autoimmune diseases, biomarker, fecal microbiota transplantation, precise therapy

The incidence of metabolic and autoimmune diseases is gradually increasing worldwide. Gut microbiota play an important role in the occurrence and development of metabolic diseases (e.g. obesity, type 2 diabetes, non-alcoholic fatty liver and so on) and autoimmune diseases (e.g. autoimmune liver disease, rheumatoid arthritis, and systemic lupus erythematosus). Targeting the gut microbiota for these diseases prevention and treatment is particularly attractive since it can use some approaches with high safety profiles and low risk of severe adverse effects, like probiotics, prebiotics, synbiotics, fecal microbiota transplantation (FMT), and antibiotics. Now, some studies have shown that regulating gut microbiota can prevent and treat these diseases. However, it is still necessary to understand the mechanism of the regulation of the gut microbiota in the prevention and treatment of these diseases and to find the most appropriate treatment methods.

This current Research Topic brings seven articles together presenting and summarizing the most recent research updates, which give us a better understanding of new potential diagnostics and therapeutics for some metabolic and autoimmune diseases on based on gut microbiota.

With the increasing popularity of the gut-liver axis concept, the concept of gut treatment of liver disease has recently been proposed. Wang et al. emphasized the important role of gut microbiota and its metabolites in the pathogenesis and development of autoimmune liver diseases including autoimmune hepatitis, primary biliary cirrhosis, primary sclerosing cholangitis as well as metabolic liver disease such as non-alcoholic fatty liver disease, cirrhosisits and its complications, and liver cancer from the perspective of immune mechanism. Furthermore, regulating gut microbiota may be novel and effective treatment for these diseases.

Rheumatoid arthritis (RA) is a chronic autoimmune disease that primarily affects the joints. Zhao et al. emphasized the important impact of gut microbiota dysbiosis in the pathogenesis of RA, and the potential of gut microbiota-related biomarkers for diagnosing RA. At the same time, therapeutic modulation of the gut microbiota and impact of gut microbiota on the efficacy of RA drugs were comprehensively summarized.

Alopecia areata (AA) accounts for the autoimmune disorder mediated by T cells, whose prognostic outcome cannot be predicted and curative treatment is unavailable at present. Kang et al. emphasized that gut microbiota has a critical effect on AA genesis and development, and their biomarkers may potentially be used as earlier diagnosis and therapeutic targets. Meanwhile, FMT may be an entirely new approach for treating AA.

For efficacy and safety of FMT in the prevention and treatment of autoimmune diseases, Zeng et al. found that the application of FMT in the treatment of six types of autoimmune diseases including type 1 diabetes mellitus, systemic sclerosis, ulcerative colitis, pediatric ulcerative colitis, crohn’s disease, and psoriatic arthritis is effective and relatively safe by meta-analysis, and it is expected to be used as a method to induce remission of active autoimmune diseases.

Diabetic retinopathy (DR) is one of the most prevalent metabolic and blinding eye diseases in ophthalmology. Liu et al. demonstrated that there was a potential causal relationship between some gut microbiota taxa and DR, which highlights the association of the “gut-retina” axis and offered new insights into the gut microbiota-mediated mechanism of DR.

Obesity has become a public health problem worldwide. Wu et al. found inulin supplementation could accelerate body weight loss in obese mice by increasing gut bacteria *Alistipes* and serum metabolite indole-3-acrylic acid level.

Although the regulation of gut microbiota is gradually widely used in the prevention and treatment of metabolic and autoimmune diseases, there is still a lack of effective methods. Therefore, researcher must focus on how to accurately regulate gut microbiota. In order to promote the development of precision metabolic and autoimmune diseases therapy based on gut microbiota, Cai et al. proposed some future research directions: (1) Identifying which types of gut bacteria are missing or overgrowing to cause a disease; (2) Determining which types of bacteria are responsible for the drug’s therapeutic effect; (3) Using gene editing techniques to target and silence the expression of disease-causing genes in certain gut bacteria; (4) Identifying metabolites and components of intestinal bacteria that are implicated in disease.

Overall, this Research Topic provides readers with new ideas for the diagnosis and treatment of metabolic and autoimmune diseases on based on gut microbiota. We could already understand from these works that the advancement of research has promoted the understanding of the role of gut microbiota in metabolic and autoimmune diseases pathogenesis. Furthermore, these works provide several insights in gut microbiome-based precision therapies while prospecting the future directions. We look forward to more new studies to illustrate the mechanisms underlying the effect of gut microbiota on promoting metabolic and autoimmune diseases occurrence, with the focus on key pathways such as bacterial dysbiosis, leaky gut, bacterial metabolites, and microorganism-related molecular patterns. In a word, it is hoped that the clinical application of gut microbiota regulation therapy brings overall health to patients with metabolic and immune diseases.

## Author contributions

YK, PR, XKu, LL and XKa wrote and revised this article. YK and YY co-edit the Research Topic. All authors made a substantial, direct, and intellectual contribution to this work and approved it for publication.

## Acknowledgments

We greatly appreciate the contributions to this Research Topic by all authors and reviewers. We thank all the guest associated editors of the Research Topic, and the editorial board of the journal of Frontiers, for their support.

## Conflict of interest

The authors declare that the research was conducted in the absence of any commercial or financial relationships that could be construed as a potential conflict of interest.

## Publisher’s note

All claims expressed in this article are solely those of the authors and do not necessarily represent those of their affiliated organizations, or those of the publisher, the editors and the reviewers. Any product that may be evaluated in this article, or claim that may be made by its manufacturer, is not guaranteed or endorsed by the publisher.

